# Investigation of the relationship between age and the angle of aortic insertion on the left ventricle using 3D MRI

**DOI:** 10.1186/1532-429X-14-S1-P77

**Published:** 2012-02-01

**Authors:** Raphael D Hazel, Simcha Pollack, Nathaniel Reichek

**Affiliations:** 1 Singapore BioImaging Consortium, BioMedical Sciences Institute, Singapore, Singapore; 2 Research and Education Foundation, St Francis Hospital, Roslyn, NY, USA

## Summary

There is a gradual increase in the insertion angle of the aorta on the left ventricle with increasing body mass index, however the increase of this angle with age in non-obese subjects is not well understood.

## Background

The aortic insertion angle (IA) is generally believed to increase with age, but this relationship has not been verified. The objective of this study was to determine the relationship between the IA of the aorta and demographic variables in humans from 3D MRI.

## Methods

Thirty four volunteers (17 males) with an average age of 53 ± 17 years were consented for this study after IRB approval. Images were acquired on a 1.5 Tesla Siemens Avanto (Siemens Medical Systems, Malvern, PA) MRI scanner with an 8 channel cardiac coil and 3D SSFP pulse sequence. Images were acquired in diastole by a delayed trigger. Sequence parameters were TR/TE = 319.5/1.3 ms, Flip Angle = 66°, FOV = 250 x 360 mm, matrix 256 x 180, slice thickness 4 - 6 mm. A 3D volume of 20-40 axial slices covering the LV and ascending aorta was acquired in a single breath-hold. In addition, in one subject, 3D volumes at each of 22 phases of the cardiac cycle were obtained. Images were interpolated to produce a 3D matrix with a spatial resolution of 1.40 mm3. The IA was defined as the angle between a line connecting the apex and the center of the mitral annulus, and the line through the center of the aortic valve and the center of the aortic lumen beyond the sinotubular junction. IA measurements were made with in-house software (Matlab, the MathWorks, Natick, MA) and validated with phantom images. Each IA is the average of 5 independent measurements.

## Results

There is a strong correlation overall between IA and BMI (r = 0.61, p < 0.001). However, in non-obese subjects, IA appears to increase with age (r = 0.55, p < 0.001). There is a weak correlation between IA and weight (r = 0.45, p = 0.008), but no significant correlation between IA and height or gender or body surface area.

## Conclusions

The insertion angle of the aorta on the left ventricle increases with body mass index.

## Funding

St Francis Hospital, 100 Port Washington Blvd, Roslyn, New York.

**Figure 1:**
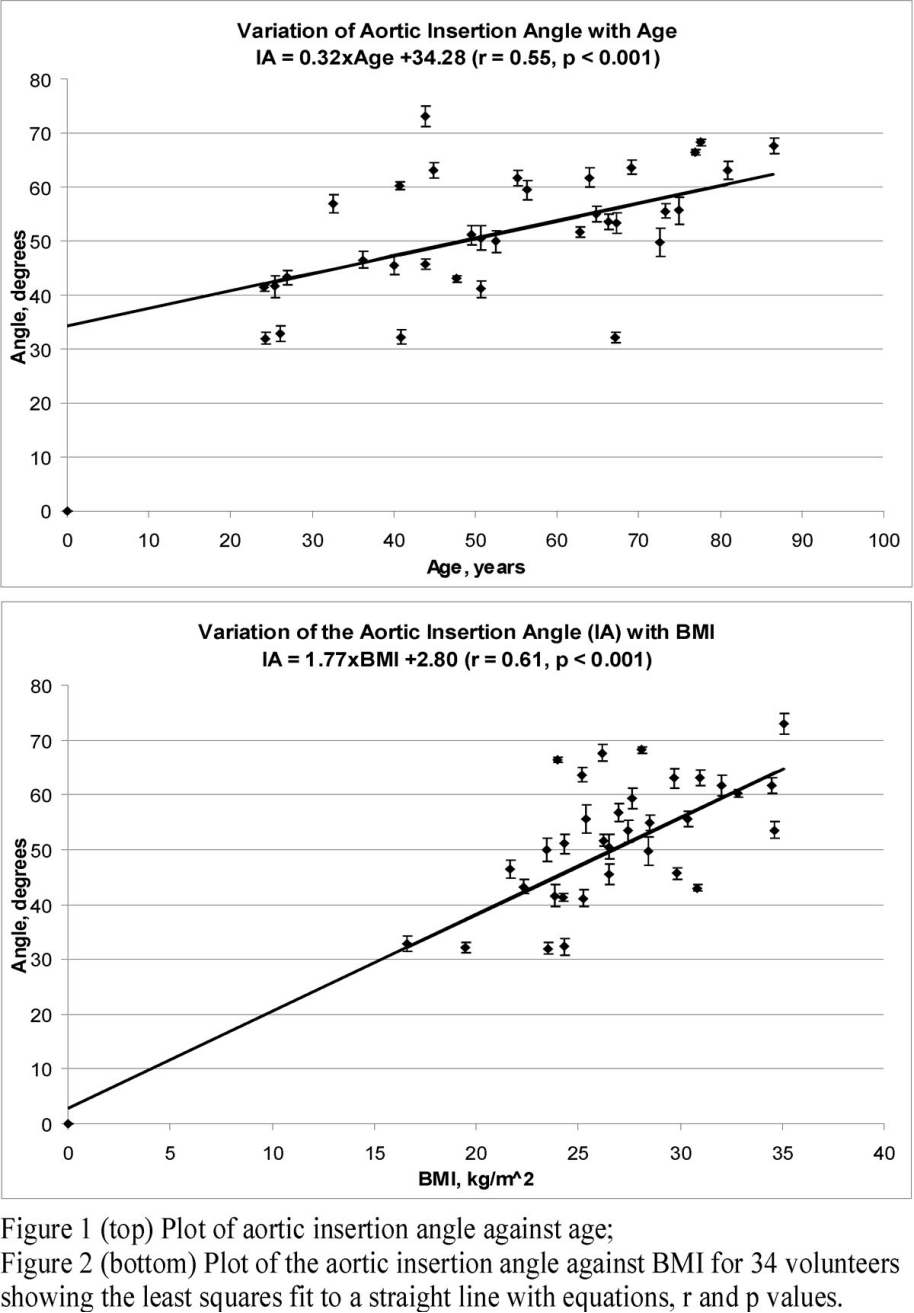
Precise measurement of aortic insertion angle using 3D MRI.

